# Prenatal Diagnosis of Anterior Urethral Valve: A Case Report

**DOI:** 10.7759/cureus.99317

**Published:** 2025-12-15

**Authors:** Clémence Vanden Berghe, Caroline Delforge, Aude Tessier, Roland Polet, Emmanuel Bollue

**Affiliations:** 1 Obstetrics and Gynecology, Cliniques universitaires Saint-Luc UCLouvain, Bruxelles, BEL; 2 Obstetrics and Gynecology, CHU UCL Namur Site De Sainte-Elisabeth, Namur, BEL; 3 Genetics and Fetal Pathology, Institut de Pathologie et de Génétique, Charleroi, BEL

**Keywords:** anterior urethral valve, lower urinary tract obstruction, prenatal diagnosis, prenatal intervention, prenatal ultrasound

## Abstract

Anterior urethral valves are a rare cause of lower urinary tract obstruction (LUTO) in the fetus. Prenatal diagnosis is primarily based on ultrasound imaging, and the condition can lead to progressive renal dysfunction and pulmonary hypoplasia secondary to oligohydramnios. Therapeutic management remains complex and lacks consensus.

We present the case of a male fetus in a 36-year-old primigravida who was referred at 16 weeks and 6 days of gestation for fetal megacystis. Morphological ultrasound revealed dilatation of the distal penile urethra, as well as megacystis and bilateral pelvicalyceal dilatation. These findings could indicate an anterior urethral valve. Two vesicocenteses were performed in succession, which revealed impaired fetal renal function with unfavorable biochemical markers. The progression to oligohydramnios and a poorly filled bladder confirmed a poor prognosis. After receiving counsel from a variety of specialists, the parents decided to proceed with the termination of the pregnancy. This procedure was performed at 19 weeks and 4 days of gestation. Although rare, an anterior urethral valve should be considered in the differential diagnosis of fetal LUTO. Prognosis depends on the timing of obstruction onset, the severity of renal involvement, and the early detection of sonographic signs. The combination of fetal imaging, urinary biomarkers, and prognostic scoring systems can aid clinical decision-making. However, further studies are needed to better define the indications for prenatal interventions and their impact on postnatal survival and quality of life.

## Introduction

Anterior urethral valves are a rare cause of lower urinary tract obstruction (LUTO) identified during the prenatal period. It may occur as an isolated anomaly or be associated with a urethral diverticulum. The underlying embryological mechanism remains unclear. Several hypotheses have been proposed, including incomplete urethral duplication, misalignment between the proximal and distal urethral segments, and an imbalance in tissue growth during urethral development resulting in residual tissue overgrowth or congenital cystic dilatation of periurethral glands [1]. They can occur in any pregnancy. In the case presented here, the patient was a 36-year-old primigravida.

The postnatal clinical presentation of an anterior urethral valve is highly variable and largely depends on the timing and severity of the obstruction. LUTO can lead to progressive bladder distension, bladder wall thickening, and subsequent bilateral uretero-hydronephrosis with renal parenchymal compression [1]. This cascade of events can impair fetal renal function and reduce amniotic fluid production, leading to oligohydramnios or even anhydramnios. The latter is a major contributor to pulmonary hypoplasia, which may result in fetal or perinatal death or severe postnatal morbidity. Some affected fetuses may develop renal failure in utero or within the first days of life, whereas others may retain normal renal function. This reflects the prognosis heterogeneity, which depends on the gestational age at the onset of obstruction, the degree of urinary tract impairment, and the extent of renal parenchymal damage [1].

The differential diagnosis of LUTO encompasses a broad spectrum of conditions and should include both common and rare causes of bladder outlet obstruction. The most frequent diagnoses include posterior urethral valves, urethral atresia, and urethral stenosis, which are the primary causes of prenatally detected infravesical obstruction. Less common causes to consider include Prune Belly syndrome, which is characterized by deficient abdominal musculature, urinary tract dilation, and cryptorchidism; meatal stenosis; mid-urethral hypoplasia; obstructive ureterocele; megacystis-microcolon-intestinal hypoperistalsis syndrome (MMIHS); and megacystis-megaureter syndrome. In addition, chromosomal syndromes, such as trisomy 21 or 18, may be associated with obstructive urinary anomalies [2].

Ultrasound features play a key role in diagnosis. An anterior urethral valve may present as penile edema, urethral dilatation, or the presence of diverticula [1]. Posterior urethral valves typically manifest as a dilated bladder with the classic “keyhole sign” reflecting a dilated proximal posterior urethra [1,2]. In severe forms of LUTO, regardless of etiology, findings may include significant bladder distension, hydronephrosis, signs of renal dysplasia, and oligohydramnios [3,4].

Previous academic literature indicate that patients with anterior urethral valves generally have a better renal outcome than those with other forms of LUTO, particularly posterior urethral valves. However, the prognosis remains variable. This condition can lead to azotemia, vesicoureteral reflux, recurrent urinary tract infections, chronic renal failure, and, in severe cases, death [5].

## Case presentation

The patient was a 36-year-old primigravida with no significant personal or family history of medical or surgical conditions. She was an active smoker, and her blood type was O Rhesus-positive.

The pregnancy progressed uneventfully until the second trimester. Non-invasive prenatal testing yielded normal results and confirmed a male fetus. The first-trimester ultrasound showed normal anatomical findings and normal biometric measurements, including a crown-rump length of 55 mm and a nuchal translucency of 1.2 mm. However, at 16 weeks and 5 days of gestation, during a routine prenatal consultation with her private obstetrician, an enlarged fetal bladder was detected on ultrasound.

**Figure 1:**
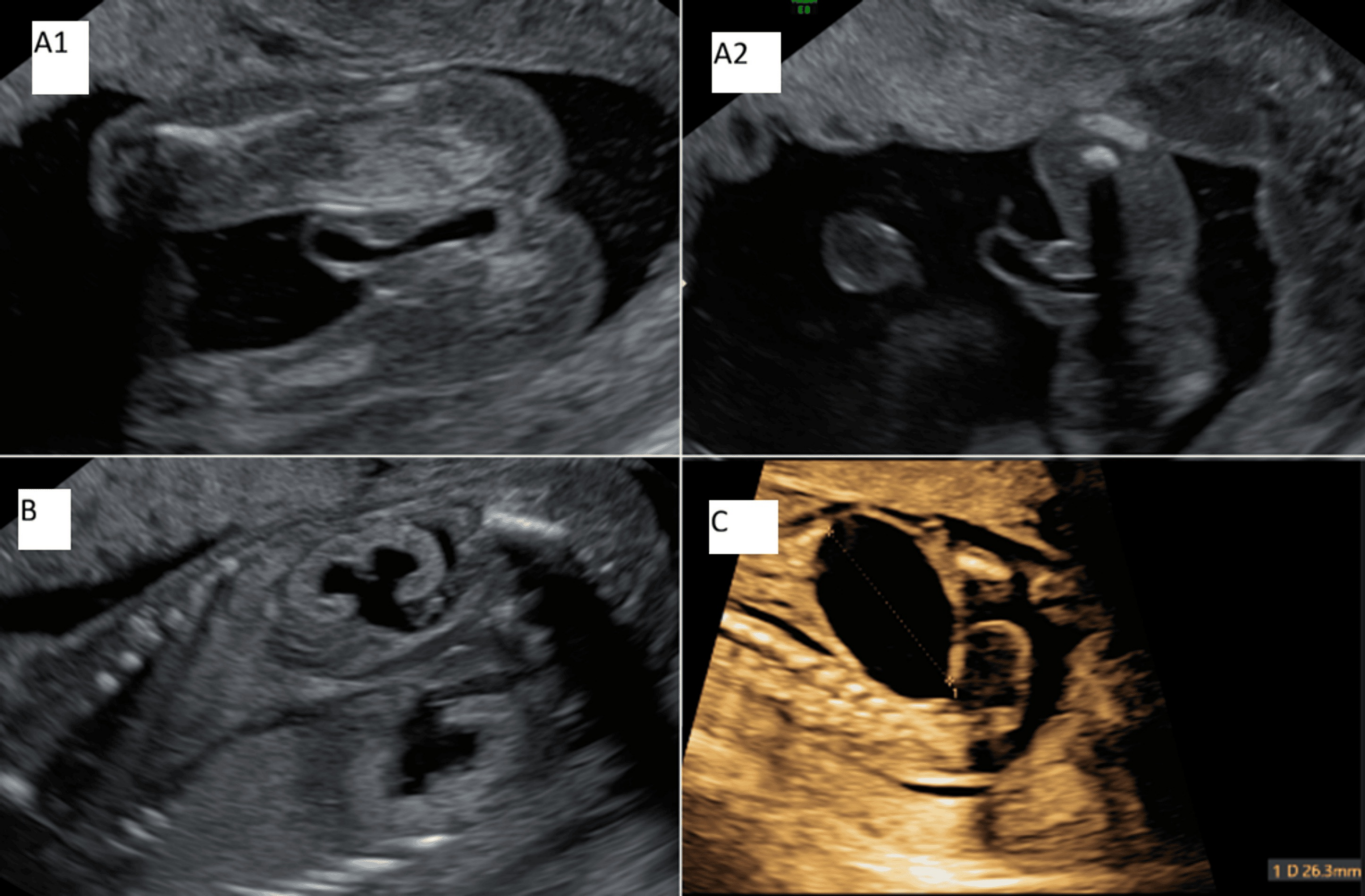
Ultrasound images of the fetus at 16 weeks and 6 days of gestation. (A1, A2) Penile urethra dilated to its distal end. (B) Bilateral hydronephrosis and hyperechoic kidneys. (C) Fighting bladder.

The patient was therefore referred to our institution for a specialized morphological ultrasound at 16 weeks and 6 days of gestation due to suspected fetal megacystis. The examination revealed dilatation of the fetal urethra extending to its distal end. A distended urinary bladder consistent with obstructive megacystis was observed, along with bilateral pelvicalyceal dilatation. The amniotic fluid index was at the lower limit of the normal range; however, the deep vertical pocket measurement was not recorded. The detailed morphological ultrasound confirmed a male fetus with findings suggestive of an anterior urethral valve (Figure 1). 

The patient was subsequently referred to a university hospital for a second opinion, amniotic fluid aspiration, and a discussion of potential vesicoamniotic shunt implantation. She was evaluated in the antenatal diagnostic unit at 17 weeks and 3 days of gestation. At that time, ultrasound revealed persistent megacystis with bilateral pelvicalyceal dilatation measuring 5.2 mm on the right and 5.38 mm on the left. The fetal kidneys appeared hyperechogenic without evidence of cysts. Urethral dilatation extending to the penile urethra was observed again, supporting the suspicion of an anterior urethral valve. The amniotic fluid volume was reduced, with a deep vertical pocket measuring 3.3 cm.

Amniocentesis allowed rapid aneuploidy testing, which confirmed a male karyotype and excluded trisomies 13, 18, and 21. Molecular karyotyping similarly confirmed a normal male profile with no pathogenic copy number variations or significant regions of homozygosity (>10 Mb), indicating the absence of unbalanced genomic abnormalities within the limits of the test and the current literature. 

Vesicocentesis was performed the same day (17+3 weeks) (Table 1). Urinary biochemical analysis yielded the following results: osmolality 274 mOsm/kg, sodium 133 mmol/L, potassium 4 mmol/L, creatinine <2 mg/L, proteinuria 0.40 g/L, and β2-microglobulin 16.31 mg/L. A follow-up ultrasound was performed at 17 weeks and 5 days, showing a persistently distended bladder with an estimated volume of 5 cm³ and stable bilateral pelvicalyceal dilatation compared to the previous examination. The amniotic fluid volume was reduced, with a deep vertical pocket measuring 3 cm. A second vesicocentesis was performed, yielding the following biochemical values: osmolality 246 mOsm/kg, sodium 117 mmol/L, potassium 5 mmol/L, creatinine <2 mg/L, proteinuria 0.40 g/L, and β2-microglobulin 14.80 mg/L. The patient had a nephropediatric consultation to explain the issues surrounding the pathology. The results from both urinary samples were considered unfavorable, indicating a poor prognosis in terms of fetal renal function.

**Table 1:** Fetal urine biochemistry (vesicocentesis).

Parameter	Patient values	Reference range (indicative of a favorable prognosis)
Osmolality	1st, 274 mOsm/kg; 2nd, 246 mOsm/kg	300-900 mOsm/kg
Sodium (Na⁺)	1st, 133 mmol/L; 2nd, 117 mmol/L	<100 to 120 mmol/L
Potassium (K⁺)	1st, 4 mmol/L; 2nd, 5 mmol/L	<8 mmol/L
Creatinine	1st, <2 mg/dL; 2nd, <2 mg/dL	<2 mg/dL
Proteinuria	1st, 0.40 g/L; 2nd, 0.40 g/L	≤0.20 g/L
β₂-Microglobulin	1st, 16.31 mg/L; 2nd, 14.80 mg/L	<4 mg/L

The patient was seen again at 18 weeks of gestation for a consultation to disclose the results. An ultrasound examination on that day showed a poorly filled fetal bladder with an estimated volume of 1 cm³. There was almost no amniotic fluid, with a deep vertical pocket measuring 1 cm. After thorough discussion and consideration, the patient and her partner opted for medical termination of the pregnancy due to the already advanced degree of renal impairment. The procedure was performed at 19 weeks and 4 days of gestation in accordance with the local legislation. In Belgium, medical termination of pregnancy can be performed at any gestational age if a severe fetal pathology is diagnosed.

**Figure 2:**
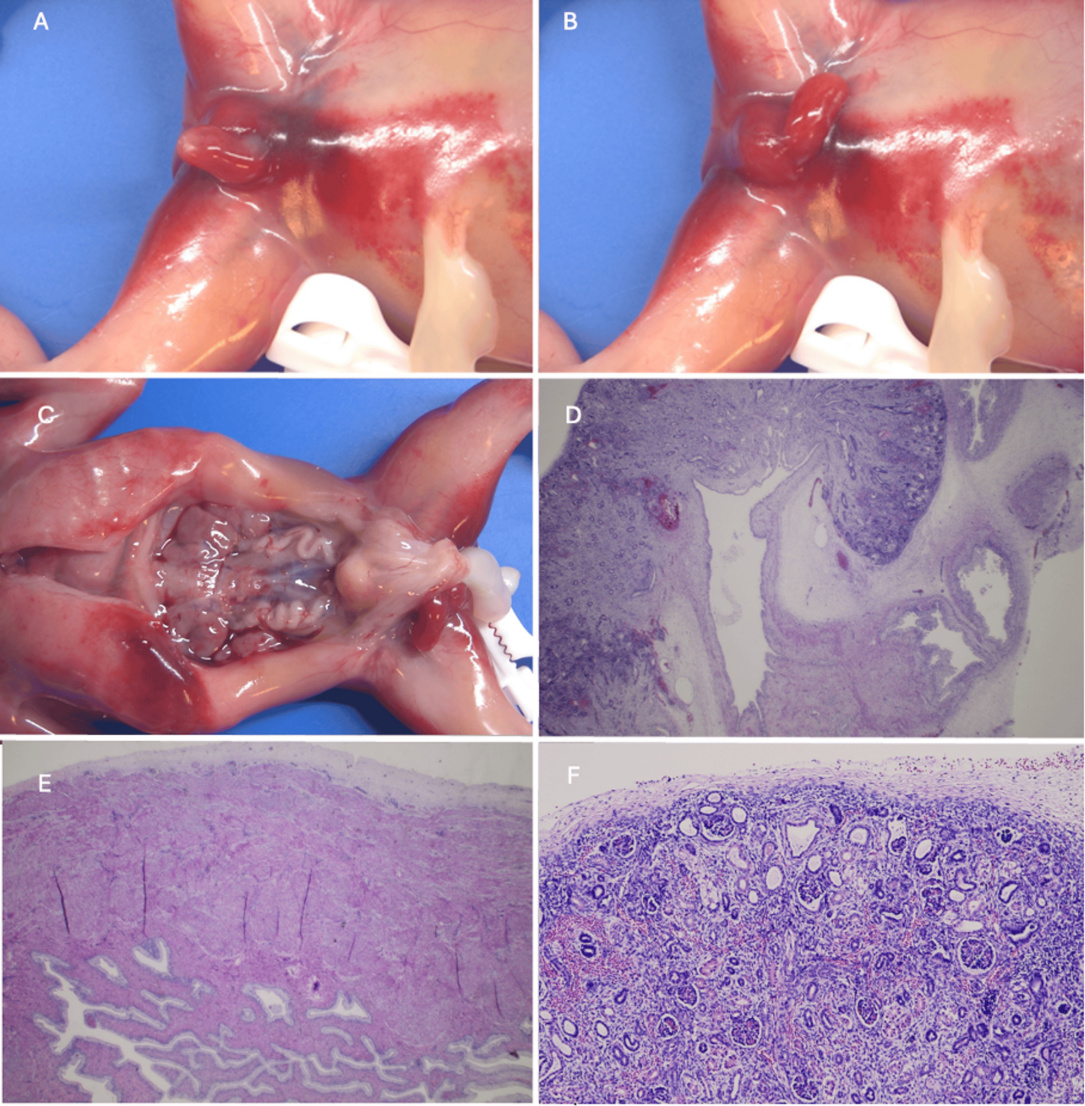
Macroscopic examination of the fetus at birth. (A, B) Dilated penis with a normal aspect of the meatus. (C) In situ pelvic view showing a megacystis and dilated ureters. (D) Loss of corticomedullary differentiation in the kidney. (E) Thickened bladder wall. (F) Small subcapsular renal cysts.

Postmortem examination showed a male fetus with normal growth. External examination found an enlarged penis without hypospadias or meatal atresia (Figure 2). The bladder measured approximately 1 cm in diameter and was unusually plump and firm, with a thick wall. Both ureters were tortuous and dilated, as was the renal pelvis. Hydronephrosis was confirmed on microscopic evaluation. Both kidneys had small subcapsular cysts, loss of corticomedullary differentiation, and a reduced metanephrogenic cortex. Tamm-Horsfall protein was found in some dilated tubes. There were no signs of anamnios (i.e., no facial dysmorphism, no lung hypoplasia). No other malformation was seen. Neuropathological examination was unremarkable. 

## Discussion

The prenatal diagnosis of LUTO presents several major challenges. The first challenge involves establishing an accurate differential diagnosis among various causes of LUTO, such as posterior urethral valves, anterior urethral valves, urethral atresia, and other rare anomalies. 

The second challenge is assessing the functional impact of the obstruction, especially its effects on renal and pulmonary development. Several approaches have been proposed to classify the severity of LUTO and predict its progression, as this assessment is crucial for guiding optimal management and supporting parental decision-making. The third challenge lies in determining the role of vesicoamniotic shunting in the management of LUTO. 

In 2013, a multicenter randomized clinical trial, PLUTO, evaluated the effectiveness of vesicoamniotic shunting compared to conservative management in fetuses diagnosed with LUTO. The results indicated a higher survival rate in the shunted group, but the potential benefit could not be conclusively demonstrated because of premature termination of the study due to insufficient recruitment. In addition, two major limitations of the study should be highlighted. First, the inclusion criteria were based on clinicians' uncertainty about the best prenatal management strategy. This is problematic given that ultrasound features used for diagnosing LUTO are highly subjective. Second, although survival rates appeared to improve, overall postnatal morbidity and mortality remained high, prompting questions about the long-term benefits of the intervention. Nevertheless, the trial suggested a potential reduction in perinatal mortality: 28-day survival was 50% (8/16) in the shunt group compared to 27% (4/16) in the control group. However, this difference was not significant. 

Finally, it is important to note that, in the PLUTO trial, shunts were placed between 16 and 22 weeks of gestation [6,7]. This is in contrast to more recent studies that suggest better renal outcomes when shunting is performed earlier in gestation [7,8]. This approach was investigated in a retrospective cohort study in which fetuses diagnosed with megacystis, defined as a longitudinal bladder diameter (LBD) > 15 mm during the first trimester, were treated using a Somatex® shunt. The study demonstrated that vesicoamniotic shunt placement is feasible during the first trimester and associated with high neonatal survival rates, primarily due to a reduction in pulmonary hypoplasia and a lower incidence of renal failure at birth. Thus, first-trimester ultrasound plays a critical role in the early detection and management of LUTO [8].

One of the key diagnostic indicators of LUTO is megacystis. However, the definition of megacystis varies among studies and remains ambiguous. In 2005, Anumba et al. defined it as an enlarged bladder that does not empty during ultrasound examination [9]. In 2013, Bornes et al. defined megacystis as LBD > 7 mm in the first trimester or one that does not empty within 45 minutes [10]. Duin et al. based their diagnosis on an LBD > 7 mm between 10 and 14 weeks of gestation combined with an inability to empty within 40 minutes [11]. Similarly, Fontanella et al. defined megacystis as an LBD > 12 mm before 18 weeks of gestation or an enlarged bladder that does not empty during a prolonged ultrasound examination of at least 40 minutes at more advanced gestational ages [12]. 

A retrospective study conducted by Fontanella et al. in the Netherlands in 2019 demonstrated that the gestational age at onset of oligohydramnios and bladder volume at the time of diagnosis are strong predictors of perinatal mortality and morbidity. Specifically, the presence of oligohydramnios was significantly associated with an increased risk of perinatal death, with an optimal predictive threshold identified at 26 weeks of gestation (area under the ROC curve: 0.95; 95% CI: 0.92-0.98; p < 0.001). Fetuses with oligohydramnios before 20 weeks and a bladder volume ≥ 5.4 cm³ were found to have a particularly poor prognosis. Based on these findings, a three-tiered classification was proposed for LUTO severity: severe LUTO, bladder volume ≥ 5.4 cm³ and/or abnormal amniotic fluid volume before 20 weeks of gestation; moderate LUTO, bladder volume < 5.4 cm³ and normal amniotic fluid volume at 20 weeks; and​​​​​​​ mild LUTO, normal amniotic fluid volume beyond 26 weeks of gestation. 

This classification correlates with a progressively decreasing risk of perinatal mortality (55%, 26%, and 9%, respectively) and severe renal insufficiency (44%, 31%, and 11%, respectively) between severe, moderate, and mild LUTOs [12].

A recent systematic review analyzing 1,049 fetuses across 20 studies further refined the identification of relevant prognostic factors. Fetal imaging, demographic data, and urinary biomarkers all contributed to the assessment of prognosis [2]. Ultrasound features associated with adverse renal outcomes include early-onset oligohydramnios or anhydramnios, increased renal echogenicity, dysplastic or cystic kidneys, and the inability of the fetus to empty the bladder. From a demographic standpoint, an earlier gestational age at delivery also correlated with poorer postnatal renal function. Regarding fetal urinary analysis, the data remain heterogeneous. Although sodium and β2-microglobulin are the most frequently studied markers, the majority of studies have not identified significant prognostic thresholds. However, two studies reported that a favorable biochemical profile (sodium < 100 mg/dL, calcium < 8 mg/dL, osmolality < 200 mOsm/kg, β2-microglobulin < 4 mg/L, total protein < 20 mg/dL) was associated with good postnatal outcomes. Conversely, high levels of β2-microglobulin (>10 mg/L) are commonly found in fetuses who progress to chronic kidney disease [2,3].

In 2016, Ruano et al. [4] proposed a four-stage classification of LUTO severity that integrates ultrasound findings, amniotic fluid volume, and urinary biochemistry to help guide treatment decisions: ​​​​​​​Stage I, normal amniotic fluid after 18 weeks, normal renal echogenicity, absence of cysts or dysplasia, and favorable urinary biomarkers (management is expectant);​​​​​​​ Stage II, oligohydramnios, hyperechogenic kidneys without dysplasia, and favorable urinary biomarkers (vesicoamniotic shunt or fetal cystoscopy is recommended);​​​​​​​ Stage III, oligohydramnios with dysplastic kidneys and/or cortical cysts and/or unfavorable urinary biomarkers (shunting may prevent pulmonary hypoplasia but not renal damage)​; and Stage IV, anhydramnios with evidence of severe renal dysplasia (prognosis is extremely poor, and invasive treatment is not recommended).

In the case presented here, the combination of urethral dilation, early oligohydramnios (deep vertical pocket 3.3 cm at 17+3 weeks, deep vertical pocket 3 cm at 17+5 weeks and 1 cm at 18 weeks), distended bladder measuring > 5 cm³ with a very low bladder-refilling rate, and poor urinary biomarker values was consistent with severe LUTO, classified as Stage III-IV according to current systems. The decision to medically terminate the pregnancy was made jointly by the multidisciplinary team and the parents, given the particularly poor renal prognosis.

International data collection is currently being carried out for a prospective multicenter observational study. The aim is to clarify the role of fetal surgery in the treatment of LUTO, including the indications and benefits of the various therapeutic approaches [7]. Emerging tools such as fetal MRI may enhance in utero evaluation of the renal parenchyma and assist in complex decision-making, although these tools are not yet routinely incorporated into clinical algorithms [2]. Further studies are needed to better understand the long-term impact of prenatal interventions, particularly in terms of renal function, urinary continence, neurodevelopment, and overall quality of life.

## Conclusions

Anterior urethral valves are a rare but significant cause of LUTO in the fetus. The broad clinical spectrum ranges from preserved postnatal renal function to severe in utero renal failure, sometimes accompanied by life-threatening pulmonary hypoplasia. The management of LUTO remains complex, primarily due to the absence of standardized guidelines and an often uncertain renal prognosis. A multidisciplinary approach combining ultrasound evaluation, fetal urinary biomarkers, and clinical context is essential to guiding therapeutic strategies and supporting parental decision-making.

## References

[1] Perlman S, Borovitz Y, Ben-Meir D (2020). Prenatal diagnosis and postnatal outcome of anterior urethral anomalies. Prenat Diagn.

[2] Pierucci UM, Paraboschi I, Mantica G, Costanzo S, Riccio A, Selvaggio GG, Pelizzo G (2024). Antenatal determinants of postnatal renal function in fetal megacystis: a systematic review. Diagnostics (Basel).

[3] Mustafa HJ, Khalil A, Johnson S (2024). Fetal lower urinary tract obstruction: international Delphi consensus on management and core outcome set. Ultrasound Obstet Gynecol.

[4] Ruano R, Dunn T, Braun MC, Angelo JR, Safdar A (2017). Lower urinary tract obstruction: fetal intervention based on prenatal staging. Pediatr Nephrol.

[5] Routh JC, McGee SM, Ashley RA, Reinberg Y, Vandersteen DR (2010). Predicting renal outcomes in children with anterior urethral valves: a systematic review. J Urol.

[6] Morris RK, Malin GL, Quinlan-Jones E (2013). Percutaneous vesicoamniotic shunting versus conservative management for fetal lower urinary tract obstruction (PLUTO): a randomised trial. Lancet.

[7] Fontanella F, Weber EC, Brinkman LA (2025). Clarifying the role of vesicoamniotic shunt in fetal medicine: three key lessons from the past and call for international registry. Ultrasound Obstet Gynecol.

[8] Strizek B, Gottschalk I, Recker F (2020). Vesicoamniotic shunting for fetal megacystis in the first trimester with a Somatex(®) intrauterine shunt. Arch Gynecol Obstet.

[9] Anumba DO, Scott JE, Plant ND, Robson SC (2005). Diagnosis and outcome of fetal lower urinary tract obstruction in the northern region of England. Prenat Diagn.

[10] Bornes M, Spaggiari E, Schmitz T (2013). Outcome and etiologies of fetal megacystis according to the gestational age at diagnosis. Prenat Diagn.

[11] Duin LK, Fontanella F, Groen H (2019). Prediction model of postnatal renal function in fetuses with lower urinary tract obstruction (LUTO)-development and internal validation. Prenat Diagn.

[12] Fontanella F, van Scheltema PN, Duin L (2019). Antenatal staging of congenital lower urinary tract obstruction. Ultrasound Obstet Gynecol.

